# Belladonna1Read before the Chicago Veterinary Society, April 9, 1900.

**Published:** 1900-04

**Authors:** L. A. Merillat

**Affiliations:** Chicago, Ill.


### BELLADONNA.[1]

By L. A. MERILLAT, V.S.,
CHICAGO, ILL.

OFFICIAL belladonna is the root or leaf of the atropa belladonna, a native plant of England and Continental Europe. In its wild state it thrives best amidst rubbish, around old ruins, along old

[1] Read before the Chicago Veterinary Society, April 9, 1900.

walls, and especially when these places are protected by shade. It will also grow vigorously when cultivated under favorable conditions in any tropical country. Atropa belladonna (commonly known as the deadly night-shade) is an herbaceous, perennial solanaceæ, growing to the height of about one metre, flowering with beautiful red bell-shaped flowers in July, and ripening its reddish berries in September.

The entire plant contains two alkaloids, occurring as salts of malic acid, chlorophyll, wax, starch, gum, albumin, lignin, and various salts; but the United States and British *Pharmacopœias* demand the use of the leaves and roots only. The leaves yield the alkaloid most abundantly, the root, fruit, and branches being next in importance in the order named. Chemically, botanically, and medicinally atropa belladonna simulates:

1. Datura stramonium (thorn apple).
2. Hyoscyamus niger (henbane).
3. Buboisia myoporoides.

Medicinally besides the above it resembles:

4. Cannabis sativa (Indian hemp).
5. Erythroxylon coca (coca).

Botanically we may add:

6. Capsicum fastigiatum (Cayenne pepper).
7. Nicotiana tabacum (tobacco).
8. Solanum dulcamara (bittersweet).

The medicinal activity of atropa belladonna depends entirely upon an alkaloid which Brandes christened atropine, and a less important alkaloid named belladonnine. Atropine is an alkaline principle with the formula $C_{17}H_{23}NO_3$. The formula for belladonnine is $C_{17}H_{23}NO_4$, containing one atom of oxygen to each molecule more than atropine.

It has been said that hyoscyamine, which has the same formula as atropine, but differs physically, is the true natural alkaloid of atropa belladonna, and that atropine is a conversion product. Chemically and medicinally atropine is identical with hyoscyamine, hyoscine, duboisine, and daturine, differing only in their physical constitution. White and Wilcox state that hyoscyamine and atropine occur together in atropa belladonna, but are not readily separated. Ladenburg regards daturine as nothing more than a mixture of atropine and hyoscyamine, while von Planta found and has since proven that daturine and atropine do not differ at all. This discrepancy is, however, of no great importance to the therapist, but we should know that:

1. Atropine is found in atropa belladonna and datura stramonium.
2. Hyoscyamine is in hyoscyamus niger, duboisia myoporoides, datura stramonium, and probably in atropa belladonna.
3. Hyoscine in hyoscyamus niger only.
4. Daturine in datura stramonium only.
5. Duboisine in duboisia myoporoides only.
6. Belladonnine in atropa belladonna only.

The preparations for veterinarians are:

1. Extractum belladonnæ radicis fluidum.
2. Extractum belladonnæ foliorum fluidum.
3. Atropinæ sulphas.
4. Homatropinæ hydrobromas.

*The fluid extract of the leaves and the root* are of uniform strength, so that it does not matter which of these two preparations is selected. The fresh leaves and roots should contain from 0.2 per cent. to 0.6 per cent. of atropine, the average being about 0.4 per cent. In regard to the strength of fluid extracts, if the product is pure and reliable, it should contain 0.004 grammes of atropine to the gramme—*i. e.*, each minim should contain about $\frac{1}{250}$ grain, and each fluidounce 1.8 grain of atropine. The fluid extract of belladonna, like many prepared drugs, varies greatly in potency, some samples being entirely wanting in medicinal activity. A prominent physician prescribed sixty grammes (two-ounce bottle) of fluid extract of belladonna for a small boy, to be taken in two-minim doses every two hours. The boy misunderstood the directions, and took two teaspoonfuls at each dose, and returned in the morning for more medicine, feeling none the worse for the error. A horse drenched with one pint (280 grammes) worked the following day without apparent inconvenience. When we take into consideration the "powerful" as well as the reliable effect of atropine itself, it is essential to demand some assurance of reliability in procuring fluid extracts which the above instances show conclusively that it is not always reliable.

*The Sulphate of Atropine.* The atropine sulphate is a very bitter, nauseating crystalline powder, obtained by dissolving atropine in dilute sulphuric acid. It is soluble in 0.4 parts of water and in about 6 parts of alcohol. Its formula is $(C_{17}H_{23}NO_3) = 2H_2SO_4$.

*The hydrobromate of homatropine* is a white crystalline substance prepared by heating atropine $(C_8H_{15}NO)X$ with oxytoluic acid in the presence of hydrochloric acid, and then neutralizing the product with hydrobromic acid. It was introduced into human ophthalmic practice about 1890, and is now coming into general use as a substitute for atropine as a mydriatic.

It is very expensive, and will be excluded from the veterinary pharmacy for some time. It is soluble in ten parts of water and in 133 parts of alcohol.

*Physiological Action of Belladonna.* Externally, if applied with substances which are absorbed into the skin, or if applied to a wound or mucous membrane, belladonna will paralyze the terminals of sensory, secretory, and motor nerves. It has a special action as a depressant to sensory nerves, and, when there is pain, is therefore an anodyne. It will dilate the capillaries and lessen the secretions of the skin and mucous membranes to which it is applied.

Internally its action is probably more complex than any of the more common medicines. Although it really has but one action, the secondary effects as a result are complicated to the extreme. This one action is that of paralyzing the periphery of the nervous system, to which might be added, *especially of secretory nerves.* The sensory and motor also share in the depression, but not to the same extent as the secretory nerves. As a result of this depression, the following phenomena is observed :

1. By depressing secretory nerves the whole aerial mucosa is markedly desiccated. The extreme dryness of the throat in human patients is a manifestation of what belladonna does to the whole respiratory mucosa. The secretion of milk is greatly diminished, and in some cases entirely arrested. Sudation is greatly depressed or even suspended, and as a consequence the skin becomes dry. The intestinal secretions are only temporarily suspended, if at all. The action of belladonna on the intestinal secretions is not well demonstrated, but it is not disputed that the effect upon the intestinal secretion is less than upon other secretions. Its action upon the renal secretion is somewhat uncertain on account of the depressing action upon the sudoriparous glands. The function of the skin being suspended, belladonna may as a result actually increase the activity of the kidneys. On the salivary secretion the action is decided, the parotid and submaxillary glands sharing specially in the depression.

2. By paralyzing inhibitory nerves the heart's action is augmented. Both the number and force of the heart-beats are increased, but more especially the former. This action of belladonna is due to paralysis of the periphery of the vagus, which inhibits the heart's action. By paralyzing the periphery of the splanchnics, belladonna augments the intestinal activity, especially peristalsis, the intestinal secretion sharing slightly from this indirect stimulation. The first effect of the drug upon the secretion of the bowels, as already stated, is that of suppression ; but later, when the inhibitory nerves become depressed, the intestinal glands resume at least their normal activity.

3. By paralyzing motor nerves the voluntary muscles become paralyzed, but this effect is noticed only in the later stages of belladonna poisoning. In ordinary medicinal dosage the effect on voluntary muscles may be said to be negative, but upon involuntary muscles its action is marked, and upon this action the real value of belladonna as a curative agent largely depends. It paralyzes the vasomotor constrictor nerves, especially of the capillary system, and so increases their lumen as to almost produce a pathological condition. The surface of the body becomes reddened and engorged with blood, and the mucous membranes are affected likewise. The organism is brought into a state that eminently resembles the first stage of a fever or an inflammatory disease—*i. e.*, augmented heart's action with heat, redness, engorgement, and dryness of the surface of the body. The capillaries of the lungs being similarly affected, the respiratory area is increased. More blood is oxidized and more $CO_2$ is eliminated. With the heart stimulated into increased activity, the capillaries of the great respiratory area active and engorged, belladonna acts as a powerful stimulant to the respiratory function —a feature possessed by no other drug. The periphery of the third nerve is paralyzed, and thus the elastic fibres of the iris contract and dilate the pupil. It was once said that atropine dilated the pupil by stimulating the sympathetic filaments of the iris, but as this theory has never been proven, and since it is difficult to conceive how a drug could depress one nerve and at the same time and under the same condition stimulate another, it is a statement that deserves no serious consideration. When the third nerve is cut the pupil dilates precisely as it does when paralyzed with atropine, therefore the mydriatic action of belladonna is easily comprehended.

4. By paralyzing sensory nerves belladonna becomes an analgesic ; but in this connection it is inferior to opium. When applied locally in a more or less concentrated form it does relieve pain, but internally it does not rank high as a pain-killer. It does, however, have a soothing effect upon the urinary tract and probably upon the generative organs.

*Brain and Spinal Cord.* The only medicinal action that belladonna has upon the brain and spinal cord is its influence on the respiratory and vasomotor centres.

SUMMARY. 1. Belladonna is a cardiac stimulant, increasing particularly the number of heart beats.

2. It arrests secretions of the skin (antihidrotic); the mucous membrane, especially the bronchial; the salivary glands (antisialagogue); and of the mammæ (antigalactagogue).

3. It does not have a marked effect on intestinal secretion, only temporarily suspending it.

4. It slightly augments peristalsis, but sufficiently to warrant its use for this purpose.

5. It stimulates the respiratory function, and in fact increases the capacity of the lungs.

6. It dilates the pupil (mydriatic).

7. Internally it has no marked effect on pain, except on the urino-generative tract, but ranks high as a local anodyne.

8. It dilates the capillaries and stimulates the action of the heart, and is therefore stimulant to the peripheric circulation.

*Therapeutics.* Belladonna has two general indications, viz., inflammation and passive hyperæmia. In any of the organic inflammatory diseases, or in any inflammatory condition, belladonna deserves to be considered in connection with their therapeutics. To say that belladonna is indicated in inflammation seems a very broad statement, and one which will probably be accepted unchallenged; but when the pathological process (inflammation) is carefully reviewed and the total effects of the drug carefully weighed, the statement is not so ambiguous as it seemed without consideration.

The period of inflammation in which belladonna is useful, if not almost a curative agent, is the stage succeeding the stage of engorgement—*i. e.*, the second or moist stage. Its tendency here is to correct the vascular alteration and to limit exudations, which in croupous, catarrhal, diphtheritic inflammation is precisely what is desired. To limit the exudations of bronchitis, laryngitis, or pulmonitis is no more or less than decreasing the ravages of inflammation, especially in the latter. When the inflammatory exudates gradually close the respiratory tract, it is part of the great battle to combat them; and besides, in this disease, that part of the lung that is not affected is rendered more capable of performing its function.

In these respiratory diseases belladonna also possesses the property of preventing cough, which is caused by the inflammatory secretions as well as the inflammation itself—in the former by drying secretions, and in the latter by allaying the irritation. In pleuritis it should prevent hydrothorax, although I cannot substantiate the statement from experience, as I have never administered it with this end in view.

In secondary nephritis occurring along the course of infectious diseases, especially when the condition has been aggravated by the liberal use of diuretics, belladonna has a beneficial influence, and also in cases of metritis and cystitis. In these diseases belladonna and saw palmetto prescribed together often give flattering results.

The organic diseases in which belladonna is especially valuable are: Croupous pneumonia, catarrhal pneumonia, bronchitis, laryngitis, nephritis, cystitis, mastitis, and metritis. It is also of service in laminitis and enteritis, but no special claims can be made for it in these diseases.

In diseases of the eye or for making examinations of the posterior region of the globe, atropine at once commends itself, and when applied locally or internally it will dilate the pupil more than any other drug, and will in this way prevent the adhesion of the iris to the lens.

*Contraindications.* Belladonna is strictly contraindicated in active hyperæmia and in the first stages of any inflammatory disease. Because of its antigalactagogue effect it should not be administered to mothers suckling their young, or to milking animals, unless its action is considered more important than the milk-supply. In inflammatory diseases of the brain and spinal cord, even small doses may cause delirium, and hence should be excluded from the therapy of these diseases. It has been prescribed in tetanus, but its value, to say the most, is doubtful.

*Administration and Dosology.* The doses of fluid extract should be about 15 grammes every twenty-four hours. One gramme dose every two hours, when given over a period of three or four days, is quite sufficient. In single doses the horse will take 8 grammes twice daily, but smaller and repeated doses give better results. Atropine, which should always be given hypodermatically, may be given in 0.06 to 0.12 gramme doses twice daily.

Atropine hypodermatically may be prescribed with strychnine (0.06 grammes of each). If given intertracheally, one-half the dose is sufficient, as atropine seems to increase the action of strychnine. I have noticed repeatedly that when these drugs are given together even small doses will produce alarming symptoms of strychnine-poisoning.

The fluid extract when given for pulmonary diseases may be combined with the fluid extract of nux vomica, and for renal or generative disorders with saw palmetto and sanmetto. In hepatized lungs ammonia muriate is a useful adjunct.

Belladonna is physiologically incompatible with pilocarpine, ammonia acetate, or any diaphoretic of the stimulating variety. On first notice it seems to be incompatible with opium, but this is not the case. Its action upon the respiration is exactly opposite, but otherwise they do not antagonize each other. However, this is of no importance in veterinary practice, as there can be no urgent need of prescribing them together. It might be argued that since one is a centric and the other a peripheric paralyzant that their union might be useful in depressing the whole nervous system in painful afflictions. However true this may be in human practice, experience will soon teach a veterinarian that belladonna in the horse is not noted for its analgesic properties.

In treatment of diseases of the eye, when dilatation of the pupil is desired, atropine in a 1 per cent. solution is dropped into the eye three or four times a day, and after the third or fourth application the result will be attained, and the pupil thus dilated may remain for several days and in isolated cases for a week. On account of this feature homatropine has entirely supplanted atropine when dilatation is desired for ophthalmoscopic examinations. A 1 per cent. solution of homatropine hydrobromate will dilate the pupil in from one to two hours, and the effect will pass off quite as rapidly, thus entirely overcoming the disagreeable feature of prolonged blindness.

