# Errata

**Published:** 1900-05

**Authors:** 


### ERRATA.

IN the JOURNAL for April, 1900, page 199, should read: "It grows vigorously, when cultivated under favorable conditions, in any part of the temperate zone."

Page 200. "The sulphate of atropine. Its formula is $(C_{17}H_{23}NO_3)_2 H_2SO_4$."

Same page. "The hydrobromate of homatropine is a white, crystalline substance, prepared by heating tropine ($C_8H_{15}NO$) with"

Page 204. "Administration and Posology."

